# Localization of Neurotrophin Specific Trk Receptors in Mechanosensory Systems of Killifish (*Nothobranchius guentheri)*

**DOI:** 10.3390/ijms221910411

**Published:** 2021-09-27

**Authors:** Marialuisa Aragona, Caterina Porcino, Maria Cristina Guerrera, Giuseppe Montalbano, Maria Levanti, Francesco Abbate, Rosaria Laurà, Antonino Germanà

**Affiliations:** Zebrafish Neuromorphology Laboratory, Department of Veterinary Sciences, University of Messina, 98168 Messina, Italy; mlaragona@unime.it (M.A.); catporcino@unime.it (C.P.); mguerrera@unime.it (M.C.G.); gmontalbano@unime.it (G.M.); mblevanti@unime.it (M.L.); abbatef@unime.it (F.A.); laurar@unime.it (R.L.)

**Keywords:** killifish, *Nothobranchius guentheri*, inner ear, lateral line system, free neuromast, neurotrophin, TrkA, TrkB, TrkC, S100 protein

## Abstract

Neurotrophins (NTs) and their signal-transducing Trk receptors play a crucial role in the development and maintenance of specific neuronal subpopulations in nervous and sensory systems. NTs are supposed to regulate two sensory systems in fish, the inner ear and the lateral line system (LLS). The latter is one of the major mechanosensory systems in fish. Considering that annual fishes of the genus *Nothobranchius*, with their short life expectancy, have become a suitable model for aging studies and that the occurrence and distribution of neurotrophin Trk receptors have never been investigated in the inner ear and LLS of killifish (*Nothobranchius guentheri*), our study aimed to investigate the localization of neurotrophin-specific Trk receptors in mechanosensory systems of *N. guentheri*. For histological and immunohistochemical analysis, adult specimens of *N. guentheri* were processed using antibodies against Trk receptors and S100 protein. An intense immunoreaction for TrkA and TrkC was found in the sensory cells of the inner ear as well as in the hair cells of LLS. Moreover, also the neurons localized in the acoustic ganglia displayed a specific immunoreaction for all Trk receptors (TrkA, B, and C) analyzed. Taken together, our results demonstrate, for the first time, that neurotrophins and their specific receptors could play a pivotal role in the biology of the sensory cells of the inner ear and LLS of *N. guentheri* and might also be involved in the hair cells regeneration process in normal and aged conditions.

## 1. Introduction

Mechanosensory systems in fish include the inner ear and lateral line system (LLS), forming together the acusticolateral system, also known as the octavolateralis system. As in mammals, in fish, the mechanosensory organs are under the control of the neurotrophins, a family of growth factor acting on different neuronal subpopulations in the central and peripheral nervous systems regulating neuronal proliferation, synaptic plasticity, development, survival, growth, and differentiation [1]. Neurotrophins’ biological functions are mediated by signal-transducing systems that are initiated by their interactions with two kinds of receptors, the high-affinity Trk receptors (tyrosine kinase receptor) and the low-affinity p75 neurotrophin receptor (p75NTR). The first are transmembrane tyrosine kinase proteins, known as TrkA, TrkB, and TrkC, interacting with their substrates in a specific but not exclusive way. TrkA binds the nerve growth factor (NGF), TrkB recognizes both brain-derived neurotrophic factor (BDNF) and neurotrophin-4 (NT-4), and TrkC interacts with neurotrophin-3 (NT- 3). TrkA and TrkB can also bind, with a lower affinity, NT-3. On the other hand, the p75 receptor can bind to unprocessed or mature neurotrophin and act as Trks co-receptor [2]. Neurotrophins and their specific receptors are evolutionarily conserved and have been found, among other vertebrates, in fishes [3,4,5,6,7]. The genome of fishes contains two additional neurotrophins called neurotrophin-6 (NT-6) and neurotrophin-7 (NT-7) [8,9,10]. As in other vertebrates, neurotrophins and their specific receptors have been localized in the fish nervous system, including sensory organs. Particularly, the occurrence of neurotrophins was also demonstrated in fish inner ear and LLS [3,11,12,13,14,15,16]. Moreover, NTs and Trk receptors were also found in the zebrafish brain [17,18,19,20,21], retina [14,22], taste buds [23], and in the crypt neurons of the olfactory epithelium [24]. It was well demonstrated that the hair cells of fish inner ears and LLS neuromasts are, under a histological, anatomical, and molecular point of view, very similar to those present in the inner ear of higher vertebrates [25,26,27]. Moreover, unlike mammals, fish retain the capability of hair cells regeneration within few days after damage, including drug treatments with ototoxic antibiotics [28,29,30]. So, it is intriguing to understand the neurotrophins possible involvement in the regenerative process of the hair cells in fish in order to find ways of promoting sensory hair cell regeneration in humans [31]. Attention on sensory hair cell regeneration is raised because hair cell damage is one of the contributors to the most common form of hearing loss, sensorineural hearing loss (SNHL) [32,33,34,35,36]. One of these disease mechanisms could be due not only to the loss of inner hair cells (IHCs) but also to their IHC synapses, impaired synaptic transmission to spiral ganglion neurons (SGNs), and disrupted propagation of auditory information along the auditory nerve [37]. A form of sensorineural hearing loss is age-related hearing loss (AHL), whose prevalence is on the rise in industrialized society because of the increase in life expectancy [38]. Therefore we found it interesting to investigate the presence of neurotrophins and Trk-like proteins in the inner ear and lateral line in a model for aging studies, as a fish of *Nothobranchius* genus. Individuals of genus *Nothobranchius* are annual fishes living in East African ponds and popular in aquariums. They have a short life expectancy in the wild and captivity [39]. Because of its short life cycle, the *Nothobranchius* has become an intriguing and emergent experimental animal model in the aging investigation since the lack of short-lived models has hampered aging research in vertebrates [40]. Moreover, the ease of isolation of vertebrate aging-related genes by homology cloning makes *Nothobranchius* a suitable model to test manipulation on aging [41,42,43], together with inexpensive embryo storage and accelerated growth [44]. Particularly, two species of this genus have been studied: *N. furzeri* and *N. guentheri*. The first one has a maximum lifespan of only 3 months and offers the possibility to perform investigations thus far unthinkable in a vertebrate, such as drug screening with life-long pharmacological treatments and experimental evolution [45]. Similarly, the annual fish *N. guentheri* has a relatively short lifespan, is commercially available, easily reared in captivity and became a suitable model for aging studies [46,47]. Additionally, it offers a set of age-related biomarkers that can be employed to track the process of tissue aging [48,49]. For all the reasons mentioned above, the possible neurotrophins involvement in aging processes of the *N. furzeri* brain started to be explored. Therefore, the expression and localization of BDNF, NGF, and NT-4, NT- 6 and of their cognate receptors have been analysed in the *Nothobranchius* with a specific attention to the brain and retina [50,51,52,53,54]. We have undertaken this study in order to analyse the immunohistochemical distribution of the Trk receptors as well as of the S100 protein in the inner ear and lateral line of *N. guentheri*. 

## 2. Results

### 2.1. Histology

**Figure 1:**
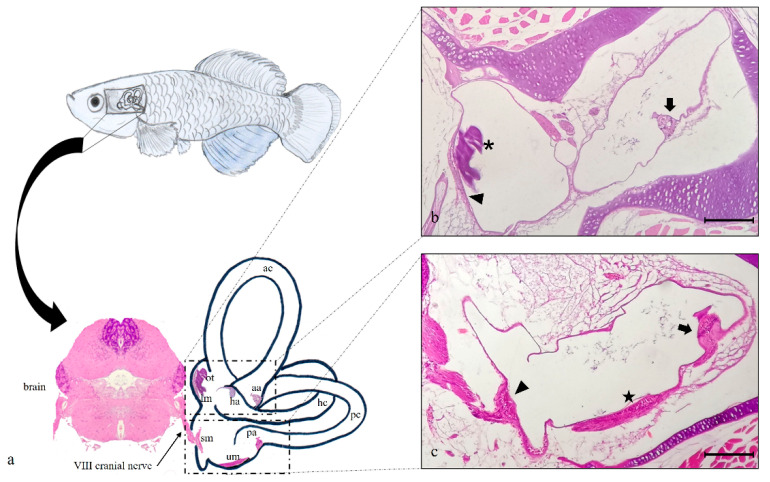
(**a**) graphical representation of *N. guentheri* inner ear that includes the three semicircular canals: anterior (ac), horizontal (hc), and posterior (pc) with their respective crista ampullaris (aa, ha, pa). Furthermore, the horizontal canal contains the macula of lagena (ml) and otholith (ot), while the posterior canal contains saccular macula (sm) and utricular macula (um). The brain from which branches off the VIII° cranial nerve that innervates the saccular macula of the posterior canal in the inner ear is shown. (**b**) Light micrographs (H&E): transversal view, the semicircular anterior canal (ac) of the inner ear, with crista ampullaris (arrow). Semicircular horizontal canal (hc) of the inner ear with macula of lagena (arrowhead) and otolith (asterisk). (**c**) Light micrographs (H&E); transversal view, the semicircular posterior canal (pc) of the inner ear, with crista ampullaris (arrow), sacculus macula (arrowhead), and utricular macula (star). Magnification 10× (**b**,**c**). Scale bars 200 µm (**b**,**c**).

**Figure 2:**
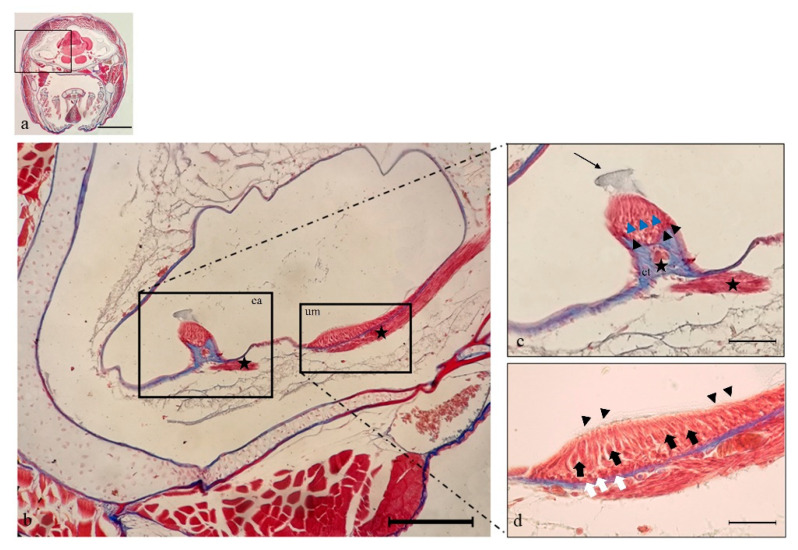
Light micrographs (Masson Trichrome with Aniline blue staining) of *N. guentheri* inner ear; transversal view. (**a**) Head. (**b**) Semicircular posterior canal of the inner ear, containing the crista ampullaris (ca) and the utricular macula (um). It is possible to observe the crista ampullaris innervation (star) and utricle macula innervation (star). (**c**) Higher magnification of crista ampullaris in the posterior canal: the connective tissue (ct) supports nerve fibers (star). The black arrowheads indicate the supporting cells, and blue arrowheads indicate the hair cells, the arrow points to the cupula. (**d**) Higher magnification of utricular macula. The portion of the utricle that forms the macula shows a sort of pouch. The sensory hair cells (black arrows) with numerous stereocilia (arrowheads) are visible. The white arrows indicate the supporting cells. Magnification 4×, scale bar 1 mm (**a**). Magnification 10×, scale bar 200 µm (**b**). Magnification 40×, scale bar 50 µm (**c**,**d**).

The inner ear of *N. guentheri* includes three semicircular canals (anterior, horizontal, and posterior), the utricle, the saccule, and the lagena. The first one represents the vestibular system, and the last one is analogous to the mammalian cochlea. The saccule and the lagena are localized in a para-medial position relative to the central nervous system. Each semicircular canal has a dilated sac at the end, representing the ampulla, with a cluster of sensory cells corresponding to crista ampullaris (Figure 1a). This one shows a cuboidal epithelium. Hair, supporting, and basal cells are present, and they form the sensory epithelium in the apical part of the crista ampullaris (Figure 2c). Inside each ampulla, there is the sensory epithelium represented by the ampullary crest of the semicircular canals. It has an orientation perpendicular to the axis of the ampulla and to the canal to which it belongs. In the utricle, saccule, and lagena, the sensory epithelium is organized into macules with large otoliths (Figure 1b). The macula of the utricle shows horizontal orientation and detects static changes in the position of the head. The macules of the saccule and the lagena are placed vertically relative to the head. The VIII cranial nerve, the cochlear vestibule nerve, penetrates the ear and flows into the ganglia. After connecting to the ganglion, the vestibular nerve divides into an upper branch that innervates the hair cells of the utricle and the ampullae of the anterior and lateral semicircular canals. The lower branch innervates the saccule and the ampulla of the posterior semicircular canal (Figure 2b).

**Figure 3:**
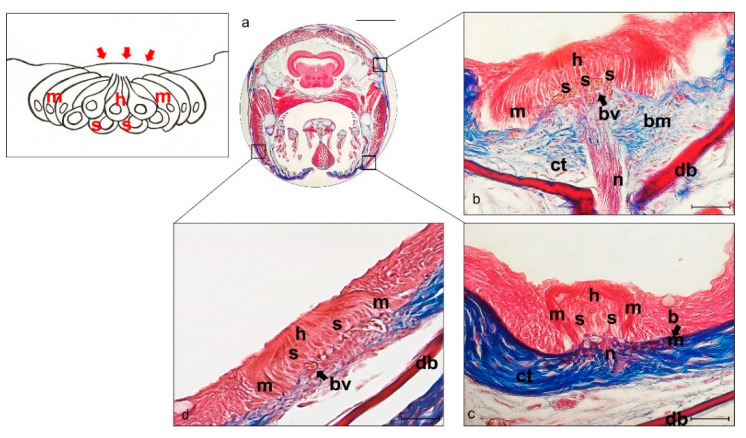
Light micrographs (Masson Trichrome with Aniline blue staining) (**a**–**d**) of lateral line system *N. guentheri* head; transversal view. (**a**) Head. (**b**) Supraorbital free neuromast. (**c**) Infraorbital free neuromast. (**d**) Iomandibular superficial free neuromast. (**b**–**d**). Free neuromast in *N. guentheri* head shows a group of hair sensory cells (h) supported by supporting cells (s) and surrounded laterally by long mantle cells (m). bv blood vessels, bm basal membrane, ct connective tissue, n nerve, db dermal bone. Magnification 4×, scale bar 1 mm (**a**). Magnification 40×, scale bar 50 µm (**b**–**d**). The insert shows a graphical representation of free neuromast, and red arrows indicate tympanic-like epithelium.

*N. guentheri* lateral line system is made up of free, superficial neuromasts that have been detected in the head and trunk. The cephalic lateral line system neuromasts of *N. guentheri* are present in the neurocranial bones located above the eye (supraorbital) and in the circumorbital bones below the eye (infraorbital and hyomandibular), on both sides of the head. Neuromasts run through in an embossed segment with incompletely ossified walls covered by a tympanic-like epithelium (insert Figure 3).

Superficial neuromasts located in the epidermis above the basement membrane were observed. The underlying dermal bone is present in the dermis. The walls of the bone canal ossify through the membrane and extend upwards from the underlying dermal bone on both sides of a neuromast (supraorbital neuromasts). These neuromasts are very large and show sensory cells surrounded by supporting and mantle cells (Figure 3b). The supporting cells are closely attached to the underlying connective, such as the mantle, to the epithelial cells. Different types of neuromasts are innervated by branches of the facial nerve: the supraorbital neuromasts are innervated by the superficial ophthalmic nerve, the infraorbital neuromasts by the buccal branch, the hyomandibular neuromasts by the hyomandibular branch.

**Figure 4:**
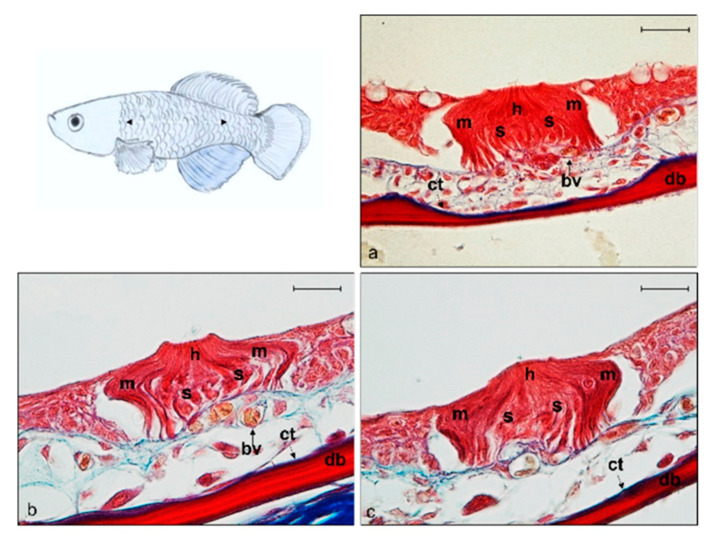
Light micrographs (Masson Trichrome with blue staining) (**a**–**c**) of lateral line system *N. guentheri* trunk; transversal view. Superficial free neuromast in *N. guentheri* trunk shows a group of hair sensory cells (h) supported by supporting cells (s) and surrounded laterally by long mantle cells (m). bv (arrow) blood vessels, bm basal membrane, ct (arrow) connective tissue, db dermal bone. Magnification 40×, scale bar 50 µm.

The lateral line system of the trunk of *N. guentheri* shows small, superficial and free neuromasts running along the trunk. They show sensory hair cells, supporting cells, and mantle cells such as head neuromasts (Figure 4a–c). 

### 2.2. Immunohistochemistry 

**Figure 5:**
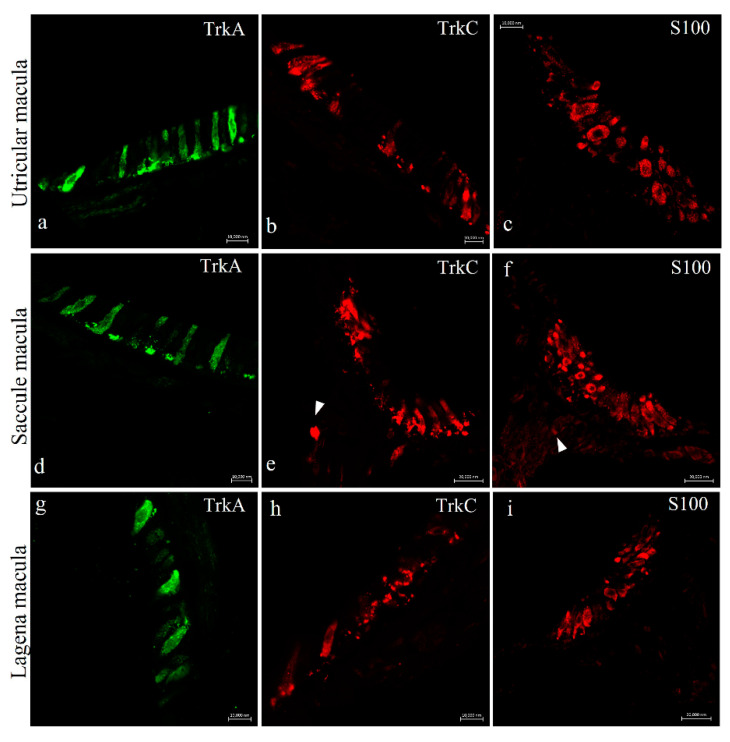
*N.guentheri* inner ear; transversal view. Immunohistochemical detection (immunofluorescence method) of Trka protein in the macula of the utricle (**a**), saccule (**d**), and lagena (**g**), of Trkc in the macula of the utricle (**b**), saccule (**e**), and lagena (**h**). Immunohistochemical detection of S100 protein in the macula of the utricle (**c**), saccule (**f**), and lagena (**i**). The utricular and saccular maculae consist of cylindrical epithelium formed by sensory hair, supporting, mantle and basal cells. Cytoplasmatic immunoreactivity for these antibodies was found in the sensory epithelial cells of the utriculus, sacculus, and lagena. Arrowheads (**e**,**f**) indicate nerves. Magnification 63× (**a**–**d**,**g**,**h**); 40× (**e**,**f**,**i**).

Immunohistochemical analysis was carried out in serial sections using single and double immunofluorescence techniques. Cellular localization of Trks was performed in the inner ear and lateral line system of *N. guentheri* using monoclonal antibodies against TrkA and TrkB and a polyclonal antibody against TrkC. To identify the positive cells immune-marked with the antibodies we used, a morpho-topographical approach based on the observation of the cellular histological features was performed. The results, using confocal laser microscopy, demonstrated that TrkA and TrkC are present in the hair cells of the utricle macula (Figure 5a,b), saccule macula (Figure 5d,e), and lagena macula (Figure 5g,h). Moreover, the immunohistochemical detection was performed in serial sections, with S100 protein utilized as a specific marker for hair cells. The TrkA and TrkC displayed a pattern of immune distribution like S100 protein, demonstrating the sensory origin of these cells (Figure 5c,f,i). Both TrkA and TrkC were localized in the cytoplasm of cylindrical cells placed in the apical part of the sensory patches of the different macules (Figure 5a,b,d,e,g,h). These cells were identified as sensory hair cells because of their morphology and localization, as well as their S100 protein immunoreactivity (Figure 5c,f,i).

**Figure 6:**
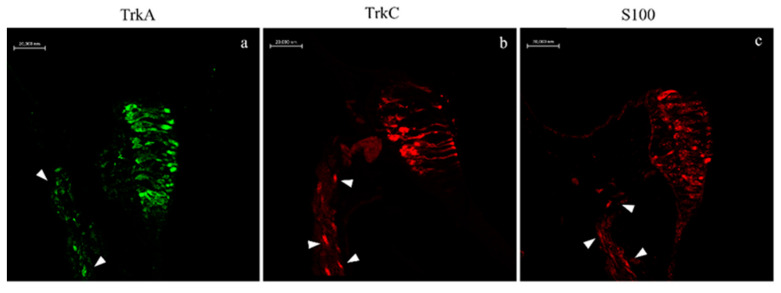
*N.guentheri* inner ear; transversal view. Immunohistochemical detection (immunofluorescence method) of TrkA (**a**), TrkC (**b**), and S100 protein (**c**) in sensory hair cells of the cristae ampullaris. The cristae ampullaris in the apical portion contain sensory hair cells, supporting cells, and basal cells. Arrowheads (**a**–**c**) indicate nerves. Magnification 40×.

An immunoreactivity for TrkA, TrkC, and S100 proteins was detected in the cristae ampullaris of the semicircular canals. In a subpopulation of hair cells, specific staining was respectively detected in the central and peripheral parts of the sensory cell cluster (Figure 6a–c).

**Figure 7:**
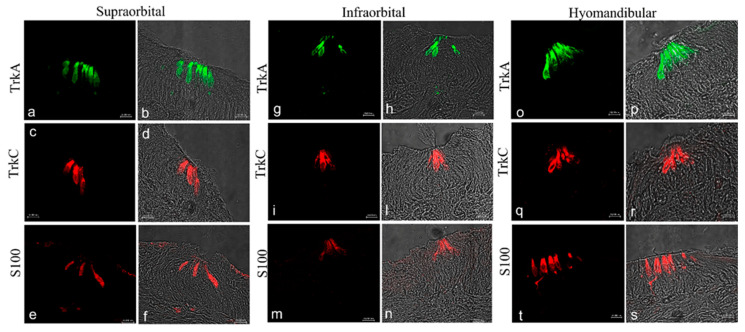
Lateral line system of *N. guentheri* head; transversal view. Immunohistochemical detection (immunofluorescence method) of TrkA, TrkC, and S100 protein. Supraorbital (**a**–**f**), infraorbital (**g**–**i**,**l**–**n**), and iomandibular free neuromast (**o**–**t**) in *N. guentheri* head show a group of sensory hair cells supported by supporting cells and laterally surrounded by long mantle cells. The hair cells in supraorbital, infraorbital, and hyomandibular free neuromast displayed cytoplasmatic immunoreactivity for TrkA, TrkC, and S100 protein. Magnification 40×.

The results obtained with confocal immunofluorescence displayed that TrkA, TrkC, and s100 antibody marked cells were located in the central-apical portion of the neuromasts, corresponding to hair cells (Figure 7a–s). Specific staining to this antibody in sensory hair cells was found in supraorbital (Figure 7a–f), infraorbital (Figure 7g–i,l–n), and hyomandibular (Figure 7o–s) of the head. 

**Figure 8:**
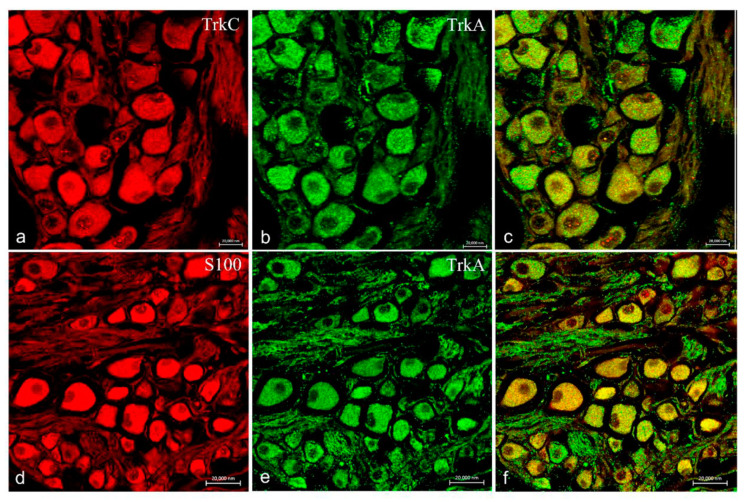
Ganglia of the VIII cranial nerve of *N. guentheri* head; transversal view. Immunohistochemical detection (immunofluorescence method) of trkA, trkC, and S100 protein. Immunoreactivity displayed to TrkA (**b**,**e**), TrkC (**a**), and S100 (**d**). Colocalization view (**c**,**f**) Magnification 40×.

**Figure 9:**
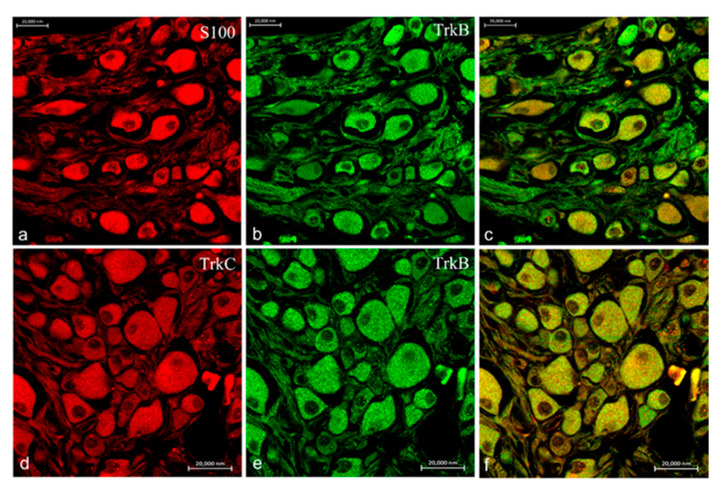
Ganglia of the VIII cranial nerve of *N. guentheri* head; transversal view. Immunohistochemical detection (immunofluorescence method) of TrkB, TrkC, and S100 protein. Immunoreactivity displayed to TrkB (**b**,**e**), TrkC (**d**), and S100 (**a**). Colocalization view (**c**,**f**). Magnification 40×.

Moreover, ganglia of the VIII cranial nerve showed an intense and specific immunoreactivity for TrkA (Figure 8b,e), TrkB (Figure 9b,e), TrkC (Figure 8a and Figure 9d) as well as S100 protein (Figure 8d and Figure 9a). No immunoreaction for TrkB receptor was found in any structure of the inner ear and lateral line system of *N. guentheri*, with particular attention to the neuroepithelium.

## 3. Discussion

The *Nothobranchius* became an intriguing and emergent experimental animal model in the aging investigation due to its short lifespan cycle. For this unique characteristic, these fish are considered a sort of bridge between mouse and *Caenorhabditis elegans,* representing two models widely utilized in biomedicine research. Specifically, the mouse is phylogenetically similar to humans but has a long life expectancy, and *C. elegans* has a short life span but is less close to us in evolutionary terms [40]. Moreover, the *Nothobrachius* represents a good model for inexpensive embryo storage, accelerated growth, and expression of aging histological and behavioral biomarkers, and easy isolation of vertebrate aging-related genes by homology cloning [45]. *Nothobranchius* is a suitable model to test manipulation on aging and study the evolution of aging-related genes by testing the effects of natural selection. Particularly, two species of this genus have been studied: *N. furzeri* and *N. guentheri*. The first one has a maximum lifespan of only 3 months and offers the possibility to perform investigations thus far unthinkable in a vertebrate, such as a drug screening with life-long pharmacological treatments and experimental evolution [45]. Similarly, the annual fish *N. guentheri* has a relatively short lifespan, is commercially available, is easily reared in captivity, and has become a suitable model for aging studies [46,47]. Additionally, it offers a set of age-related biomarkers that can be employed to track the process of tissue aging [48,49]. In this study, we demonstrate for the first time the localization of Trk receptor proteins in the inner ear and lateral line system of *N. guentheri*. Moreover, in this research report, the S100 protein was used to identify the sensory cells present in the mechanoreceptor organs. S100 proteins are a large subfamily of EF-hand Ca^2+^-binding proteins localized in the cytoplasm and/or nucleus of a wide range of cells, participating in the regulation of intracellular Ca^2+^ homeostasis as a trigger or activator proteins. This protein was previously utilized in zebrafish as a specific marker for hair cells of the inner ear sensory patches and sensory cells of lateral line neuromast [23,55,56,57,58]. Moreover, the S100 protein expression has been demonstrated in the glial cells and neurons of zebrafish and different areas of *N. furzeri* central nervous system [51,59]. In this investigation, the localization of neurotrophin-specific Trk receptors was observed in the sensory cells of the killifish mechanosensory systems made up of the inner ear and lateral line system (LLS). In fish, the lateral line system consists of superficial neuromasts (SN) and canal neuromasts (CN), partially deriving from the neural crest [60]. The lateral line system can significantly diversify between different fish species, and the distribution of SN and CN shows a high degree of variability among these animals. Surface-feeding fishes detect surface waves caused by distressed insects that have fallen into the water with their cephalic LLS [61,62]. This could be in line with our results, demonstrating bigger neuromasts in the cephalic lateral line system than trunk LLS neuromasts in *N. guentheri*. The latter are innervated by the vagus lateral branch [63]. Finally, our results demonstrate that in *N. guentheri,* LLS is made up of free and superficial neuromasts lifetime and not only during larval and juvenile stages such as in other species (e.g., zebrafish).

Particularly, we found a specific immunoreaction for TrkA and TrkC in the hair cells of the macula of the utricle, saccule, and lagena, in the crista ampullaris of the inner ear and the central cluster of hair cells of lateral line neuromasts. It is well known that neurotrophins and their specific Trk receptors are required for neuronal survival of the central and peripheral nervous system, including sensory organs [14,22,23,24,56,64] and that the neurotrophins sequence is well conserved during evolution with an origin of the Trk family dated approximately 600 million years ago [4,65]. The specificity of the antibodies used was previously tested and demonstrated in the brain of *N. furzeri* using Dot-blot analysis and immunohistochemistry [51]. The obtained results of this report are in agreement with previous studies performed in different species of fish, including zebrafish (*Danio rerio*), sea bass (*Dicentrarchus labrax*), salmon (*Salmo salar*), and trout (*Salmo trutta*) regarding the localization of Trk receptors in the hair cells of mechanoreceptor tissue of fish auditory systems [11,12,13,14,16]. In recent papers from our group of investigation, the expression and localization of BDNF/TrkB system in the lateral line and inner ear of zebrafish were showed [15]. In contrast, in this study, no immunoreaction for BDNF and TrkB was observed in the hair cells of *N. guentheri*. A specific and intense immunoreaction for TrkB was found in the neurons of the acoustic ganglia demonstrating the presence of the BDNF/TrkB system in *Nothobranchius*, but not in the mechanoreceptor organs. This discrepancy might be due to a different regulation system of the biology of sensory cells in the short lifespan fish and could also be related to the short life cycle of the fish characterized by the rapidly changing of the protein expression of the sensory cells during the physiological process of hair cell regeneration.

It is well demonstrated that the neurotrophin family plays an important role in the synaptic plasticity of sensory hair cells. Particularly, the BDNF/TrkB and NT-3/TrkC systems exert a specific and important function in the regulation of the auditory system in mammals [66], supporting the maintenance of the spiral ganglion neurons in culture and suggesting their potential therapeutic use as a novel drug in sensory hearing loss disorders [67]. Mammals have a limited capacity to regenerate cells and tissue, and this activity decreases sharply during aging. In mammals, the capability to regenerate inner ear hair cells is very restricted or absent. Therefore, the loss of sensory cells in humans leads to a wide range of inner ear disorders with a permanent reduction of hearing and balance. In this way, our results demonstrate, for the first time, that also the sensory cells of the inner ear and lateral line system of *N. guentheri*, as previously observed in zebrafish [15,68,69], are very similar, from a morpho-physiological point of view, to human sensory cells. Moreover, fishes maintain the possibility to regenerate the hair cells following damage caused by a traumatic event, intense acoustic stimuli, environmental insults, and pharmacological or ototoxic chemicals treatment. Specifically, it has been well demonstrated, using zebrafish transgenic line, that the lateral line sensory cells can regenerate under experimental conditions within few days, completely restoring their functional activities [28,29,31,70,71,72,73,74,75,76,77,78]. Our results are in line with the hypothesis that neurotrophins and their receptors may play a role in the regeneration process; indeed, we observe an immunopositivity to Trks receptors in the hair cells of the LLS neuromasts and of the inner ear. For this reason, the fish auditory system and especially the mechanoreceptor organs of *Nothobranchius* for the characteristic of short life cycle might represent an excellent model to deeply analyze the molecular events linked to the regeneration process that might represent a cardinal point in the study of aging inner ear disorders.

In *N. guentheri*, the presence of TrkA and TrkC in the sensory cells of the macula as well as of the crista and in the neuromast of the lateral line system might suggest a key function of NGF and NT-3 in the complex mechanism of sensory organs homeostasis in the annual fish, such as in mammals [66]. Moreover, the presence of NGF, NT-4, and NT-6 was detected in the brain of *N. furzeri* [52,79,80], where the authors hypothesize that the NGF/TrkA system could modulate several physiological functions in the adult brain of *N. furzeri,* playing a role during aging processes. Finally, based on the obtained results, we can assume that the *Nothobranchius* could represent a suitable model to study age-related hearing loss studies continuing to investigate the role of growth factors in the biology of sensory cells within the sensory patches of sensory organs. 

## 4. Materials and Methods

### 4.1. Fish and Tissue Treatment

In this study, we used adult specimens of *N. guentheri* found dead by unknown causes (1-year-old, 1 male, and 2 females) in ornamental aquariums (freshwater, 22 °C, pH 6.8–7.0). The heads were quickly removed, fixed in 4% paraformaldehyde in phosphate-buffered saline (PBS) (AAJ19943K2, Thermo Scientific) 0.1 m (pH = 7.4) for 12–18 h, dehydrated through graded ethanol series, and clarified in xylene for paraffin wax embedding. Included tissues were then cut into 7 μm thick serial sections and collected on gelatin-coated microscope slides. Then, serial sections were deparaffinized and rehydrated, washed in distilled water, and stained with hematoxylin and eosin (H&E) (Carazzi’s Hematoxylin Nuclear staining, 05-M06012; Eosin Y 1% aqueous solution cytoplasmic staining, 05-M10002, Bio-Optica Milano s.p.a.) and Masson Trichrome with aniline blue method (04-010802, Bio-Optica Milano s.p.a.). Sections were examined under a Leica DMRB light microscope. 

### 4.2. Localization of TrkS and S100 Protein Using Single and Double Immunofluorescence Staining

To analyze the expression of different proteins in the sensory patches of the inner ear, lateral line system, and the acoustic ganglia, sections were deparaffinized and rehydrated, washed in Phosphate-Buffered Saline (PBS) 0.1 M pH = 7.4, and incubated in 0.3% H_2_O_2_ (PBS) solution for 3 min to prevent the activity of endogenous peroxidase; then fetal bovine serum (F7524 Sigma-Aldrich) was added to rinsed sections. Sections were incubated overnight at 4 °C in a humid chamber with antibodies anti-Trks and anti S100 protein that recognizes a mixture of both s100A and s100B proteins subunit (see Table 1). Moreover, to analyze the expression of different proteins in acoustic ganglia, TrkA and TrkB monoclonal antibodies were used in double-label experiments with polyclonal antibodies TrkC and S100 (see Table 1). 

After rinsing in PBS, the sections were incubated for 1 h at 4 °C with anti-Mouse IgG (H+L) Alexa Fluor 488 (Invitrogen, Waltham, MA, USA; catalog number A-21202; diluted 1:300) and anti-Rabbit IgG (H+L) Alexa Fluor 568 (Invitrogen, USA; catalog number A-21207; diluted 1:300). Both steps were performed at room temperature in a dark, humid chamber. Finally, the sections were washed, dehydrated, and mounted with Fluoromount Aqueous Mounting Medium (Sigma Aldrich, St. Louis, MO, USA). The immunofluorescence was detected using a Zeiss LSMDUO confocal laser scanning microscope with META module (Carl Zeiss MicroImaging GmbH, Göttingen, Germany), and the captured images were processed using Zen 2011 (LSM 700 Zeiss software). Each image was rapidly acquired to minimize photodegradation. Digital images were cropped, and the figure montage was prepared using Adobe Photoshop 7.0 (Adobe Systems, San Jose, CA, USA). To provide negative controls, representative sections were incubated with specifically preabsorbed antisera as described above. Under these conditions, no positive immunostaining was observed (data not shown). 

## Figures and Tables

**Table 1:** Primary Antibodies.

Primary Antibodies	Supplier	Catalogue Number	Source	Diluition	Antibody ID
S100	Dako	Z0311	rabbit	1:100	AB_10013383
TrkA (Y32Ex)	Santa Cruz Biotechnology, Inc.	sc-80398	mouse	1:100	AB_1130726
TrkB (F-1)	Santa Cruz Biotechnology, Inc.	sc-377218	mouse	1:100	AB_2801499
TrkC (798)	Santa Cruz Biotechnology, Inc.	sc-117	rabbit	1:100	AB_632560

## Data Availability

All data presented this study are available from the corresponding author, upon responsible request.
